# RASopathies and hemostatic abnormalities: key role of platelet dysfunction

**DOI:** 10.1186/s13023-021-02122-7

**Published:** 2021-12-02

**Authors:** Francesca Di Candia, Valeria Marchetti, Ferdinando Cirillo, Alessandro Di Minno, Carmen Rosano, Stefano Pagano, Maria Anna Siano, Mariateresa Falco, Antonia Assunto, Giovanni Boccia, Gerardo Magliacane, Valentina Pinna, Alessandro De Luca, Marco Tartaglia, Giovanni Di Minno, Pietro Strisciuglio, Daniela Melis

**Affiliations:** 1 Dipartimento di Scienze Mediche Traslazionali, Università degli studi di Napoli Federico II, Naples, Italy; 2 Regional Reference Centre for Coagulation Disorders, Department of Clinical and Experimental Medicine, Federico II University of Naples, Naples, Italy; 3 Department of Medicine, Surgery and Dentistry, University of Salerno, Baronissi, Salerno Italy; 4 Pediatric Unit, San Giovanni di Dio e Ruggi d’Aragona University Hospital, Salerno, Italy; 5 Clinic Pathology, San Giovanni di Dio e Ruggi d’Aragona University Hospital, Salerno, Italy; 6 Medical Genetics Division, Fondazione IRCCS Casa Sollievo della Sofferenza, San Giovanni Rotondo, Foggia Italy; 7 Genetics and Rare Diseases Research Division, Ospedale Pediatrico Bambino Gesù, IRCCS, Rome, Italy

**Keywords:** RASopathies, Noonan syndrome, Bleeding disorders, Laboratory test abnormalities, Screening surgical procedures, Abnormal platelet function, Platelet optical aggregometry

## Abstract

**Background:**

Bleeding anomalies have been reported in patients affected by Noonan syndrome. No study has been performed in patients with molecularly confirmed RASopathy. We aimed to characterize the frequency and types of bleeding disorders in patients with RASopathies and evaluate any significant association with laboratory findings.

**Patients and methods:**

Forty-nine individuals (*PTPN11*, n = 27; *SOS1*, n = 7; *RIT1*, n = 3; *SPRED1*, n = 1; *LZTR1*, N = 3; *RAF1*, n = 2; *BRAF*, n = 4; *MEK1*, n = 1; *MEK2*, n = 1), and 49 age- and sex-matched controls were enrolled. The “Paediatric Bleeding Questionnaire Scoring Key” was administered to patients and families. Laboratory screening tests including clotting factors dosing, platelet count, Prothrombin Time and Partial Thromboplastin Time, were employed both in patients and controls to characterize the bleeding diathesis. A subgroup of 29/49 patients and 29/49 controls was also tested for platelet function.

**Results:**

Regardless of the gene involved, pathological paediatric bleeding scores were recorded in 14/49 (28.5%) patients. Indeed, 7 were mutated in *PTPN11*, 3 in *SOS1*, 2 in *RIT1*, 1 in *BRAF*, and 1 in *MEK1*. Compared to patients with normal bleeding scores, those with pathologic bleeding score showed higher prevalence of splenomegaly (*p* = 0.006), prolonged aPTT (*p* = 0.04), lower levels of coagulation factor V (FV, *p* = 0.001), FVII (*p* = 0.003), FX (*p* = 0.0008) and FXIII (*p* = 0.002), higher vWAg (*p* = 0.04), and lower platelet sensitivity to Ristocetin (*p* = 0.001), arachidonic acid (AA) (*p* = 0.009) and collagen (*p* = 0.01). The presence of hematomas inversely correlated with factor V (*p* = 0.002), factor VII (*p* = 0.003), factor X (*p* = 0.002) and factor XIII (*p* = 0.004) levels, and directly correlated with platelet response to collagen (*p* = 0.02) and AA (*p* = 0.01). The presence of splenomegaly directly correlated with the presence of hematoma (*p* = 0.006), platelet response to Ristocetin (*p* = 0.04) and AA (*p* = 0.04), and inversely correlated with factor V levels (*p* = 0.03).

**Conclusions:**

Patients with RASopathies and a bleeding tendency exhibit multiple laboratory abnormalities, including platelet-related disorders. Splenomegaly is frequently detected and might be a suggestive sign for qualitative platelet dysfunction. A comprehensive clinical assessment should be carried out at diagnosis, during the follow-up and before any surgical procedures. Since there is currently no consensus on management of bleeding complications, it is important that physicians closely monitor these patients.

## Introduction

RASopathies represent one of the most common family of genetic syndromes affecting development and growth. These disorders are caused by germline mutations in genes encoding components of the Ras/mitogen activated protein kinase (MAPK) pathway, a ubiquitous and highly conserved signal transduction cascade critical to normal development with a key role in cell cycle regulation, differentiation, proliferation, apoptosis and senescence [1]. The proteins involved in these disorders include *PTPN11* and multiple members of the RAS subfamily of GTPases (*HRAS, NRAS, MRAS, RRAS, RRAS2* and *RIT1*), core components of the MAPK cascade (*BRAF, RAF1, MAP2K1, MAP2K2* and *MAPK1)*, and both positive (*SOS1, SOS2, SHOC2 and PPP1CB*) and negative (neurofibromin and *SPRED*1) regulators of Ras function. This signalling cascade controls the function of a large number of downstream effectors, including a variety of nuclear regulatory proteins involved in the control of gene expression and cell cycle progression, and several cytoplasmic effectors controlling diverse cellular processes e.g., differentiation, metabolism and survival [1].

The RASopathies include cardiofaciocutaneous syndrome (CFC, OMIM 115150), Costello syndrome (CS, OMIM 218040), neurofibromatosis type 1 (NF1, OMIM 162200), Legius syndrome (OMIM 611431), Noonan syndrome (NS, OMIM 163950), Mazzanti syndrome (OMIM 607721 and 617506), Noonan syndrome with multiple lentigines (formerly known as LEOPARD syndrome; LS, OMIM 151100) and other emerging disorders [2–7]. These diseases are characterized by a clinical overlap, with similar craniofacial, cardiac, and cutaneous features, and skeletal abnormalities, variable neurocognitive impairment, and behavioural abnormalities, hypotonia, defective postnatal growth and a variably enhanced risk of cancer. Despite their clinical similarity, each of these disorders has unique phenotypic features that likely result from a differential impact on development of the specific way of dysregulation of the pathway [2–7]. A major clinical complication of NS is the abnormally high risk of bruising and spontaneous bleeding, reported in 50–89% of patients [8]. Bleeding abnormalities are often associated with factor XI deficiency and platelet abnormalities in patients with NS. Since a significant number of NS require surgery, their tendency to bleed is a relevant concern. For instance, a duodenal hematoma has been reported as complication of endoscopic biopsy in NS [9] and argues for the early identification of subjects at risk before the procedure among affected individuals to set up the optimal management [10–20].

SHP2, the protein encoded by *PTPN11* (the gene most frequently mutated in NS) is thought to play an important role in platelet function. Indeed, SHP2 is involved in intracellular signalling, in response to a wide range of growth factors, cytokines and hormones [21]. It has also been implicated in regulating signalling from a variety of platelet and megakaryocyte receptors. Megakaryocyte/platelet (MP)-specific deletion of Shp2 resulted in macrothrombocytopenia and platelets become hyper-responsive to anti-CLEC-2 antibody and fibrinogen [22, 23]. Of note, at odds with NS patients with activating mutations of this gene and high prevalence of bleeding disorders [2, 3], patients with LS "carrying inactivating mutations" [24], do not have bleeding disorders [2, 3]. In a NS mouse model carrying a gain-of-function mutation of SHP2 a significant reduction in platelet aggregation is reported, induced by low concentrations of GPVI and CLEC-2 agonists [25]

In the current study, we systematically evaluated ex vivo a series of coagulation and platelet function variables in a cohort of molecularly characterized RASopathy patients to evaluate the prevalence of common haemostatic abnormalities in these disorders and identify possible genotype–phenotype correlations.

## Patients and methods

All the subjects were enrolled at the Clinical Genetic Pediatric Department of the University "Federico II" of Naples (Italy). In total, 49 patients (18 females, 31 males) were included in the study. Their clinical diagnosis (NS, N = 43; CFCS, N = 2; LS, N = 2; NFNS, N = 2) had molecularly been confirmed. The mean age at enrolment in the study was 10.8 years (ranging from 0 to 49 years). A sex- and age-matched control group including 49 subjects (18 females, 31 males; mean age 11.2 years, ranging from 1 to 49 years) was also enrolled. Only patients with a molecular diagnosis of RASopathy were considered. The cohort presented the following distribution: *PTPN11* (27, 53.1%), *SOS1* (7, 14.3%), *RIT1* (3, 6.1%), *SPRED1* (1, 2.0%), *LZTR1* (3, 6.1%), *RAF1* (2, 4.1%), *BRAF* (4, 8.2%), *MEK1* (1, 2.0%) and *MEK2* (1, 2.0%). All patients underwent anamnestic recall, clinical and auxological examination, and spontaneous hematomas, ecchymoses and epistaxis were individually evaluated. The “Pediatric Bleeding Questionnaire (PBQ) Scoring Key” [26] was administered to patients and families to assess the patients’ tendency to bleed and bruise. PBQ score results above 3 were considered indicative of an enhanced tendency to bleeding or bruising forming. The degree of bleeding diathesis was also evaluated by laboratory screening tests including clotting factors dosing, platelet count, Prothrombin Time (PT) which measures the integrity of the extrinsic system as well as factors common to both systems and activated Partial Thromboplastin Time (aPTT), which measures the integrity of the intrinsic system and the common component in both patients and controls, performed according to standard methods.

29 out of the 49 patients with RASopathy (13 females, 16 males; mean age 11.8 years) and 29/49 controls with no history of bleeding underwent a platelet function test. It consists in a “turbidometric platelet aggregometry”, which measures platelet aggregation in platelet-rich plasma (PRP, obtained by centrifugation of 9 volumes of freshly collected whole blood [from the antecubital vein] in plastic tubes containing one volume of 3.8% trisodium citrated and centrifuged for 15 min at 700×*g*) [10, 11]. The method is based on the detection of difference in light transmission by a photometer after adding a platelet agonist to PRP under stirring conditions (1000 rpm). Samples can be exposed to a wide range of agonists, which can give an insight into different pathways of platelet activation/aggregation; in the current study ADP, arachidonic acid (AA), collagen and ristocetin have been used. Aggregation measurements provide an aggregation index curve describing changes in the intensity of the light transmission of the PRP samples versus a comparator sample of platelet-poor plasma (PPP) obtained from the citrated blood centrifuged at 2000×*g* for 15 min after removing the supernatant PRP.

Platelet aggregation was determined in a Lumi-aggregometer (Chronolog Co., Havertown, Pa.) that also records the luminescence resulting from the interaction of released ATP (secreted simultaneously with ADP) with firefly luciferase and luciferin (Chronolume 395, Chronolog Corp., Havertown, Pa.). The apparatus was adjusted so that PRP and PFP produced 10% and 90% light transmittance, respectively. An aggregating agent or an equal volume of vehicle was added in microliter amounts to 0.5 ml of platelet suspension that had been stirred at 1000 rpm and 37 °C for 1 min. The maximal light transmittance achieved within 3 min after the addition of threshold concentrations of arachidonic acid (AA) or of 0.4 µM adenosine diphosphate (ADP) was defined as max A%. A value of ≥ 50% light transmittance (LT-50%) in response to 1.0 mM AA or of 10 µM ADP within the same time period defined platelet hyper-reactivity. EC_50_ was defined as the lowest concentration of an agent added to a platelet suspension that caused more than 50% light transmittance within 3 min in response to threshold concentrations of aggregating agents. Pathologic response in platelet suspensions from patients with RASopathies was defined as the lack of at least 50% light transmittance in response to concentrations of each aggregating agent significantly greater (*p* always < 0.05) than those needed to achieve such light transmittance in controls.

## Statistical analysis

Each numerical variable was expressed as mean ± SD. Statistical analyses were performed using SPSS package 10. Differences in the clinical and biochemical parameters between patients and controls as well as between patients with pathologic bleeding score and patients with normal bleeding score were analysed using the t-test for paired data. Genotype–phenotype correlations were performed taking into account the individual gene involved and type of mutation. Fisher exact test was used in these comparisons to assess the statistical significance of deviations. To analyse possible correlations between the severity of features and specific gene involved, Spearman’s correlation study and association study were performed. A *p* value < 0.05 was considered to be significant in all comparisons.

## Results

All patients underwent anamnestic recall, clinical examination, including auxological parameters, evaluation of hematomas, ecchymoses and epistaxis. Auxological parameters were normal for specific growth chart in 47/49 patients. Bleeding was recorded in 18/49 (36.7%) patients; hematomas and or ecchymoses were recorded in 13/49 (26.5%) patients, while epistaxis and oral bleedings were reported in 3/49 (6.12%) and 11/49 (22.4%) patients, respectively. Menorrhagia was recorded in 3/8 (37%) menstruating females.

Pathologic bleeding score (PBQ ≥ 3) was recorded in 14/49 (28.5%) patients. Significant scores included: PBQ of 3 in 11 patients; PBQ of 4, 5 and 7 in single patients each.

The degree of bleeding diathesis was also evaluated by laboratory screening tests (clotting factors dosing, platelet count, PT and aPTT assessment) in patients and compared to age- and sex-matched control group. Pathologic laboratory tests included: prolonged PT (35 patients), aPTT (16 patients); PT/aPTT (13 patients). One subject presented thrombocytopenia (platelet count below 100,000/mm^3^). 17/49 (32.6%) patients had splenomegaly. Being reported in 12/49 (24.4%) patients, factor VII partial deficiency, isolated or in combination with the deficiency of other vitamin K-dependent factors, was the most frequent coagulation factors abnormality. The optical aggregometry test was pathologic in 19/30 patients, demonstrating platelet dysfunction in 65% of RASopathy patients. Table 1 shows that the concentrations of ADP, and collagen required to cause 50% light transmittance were significantly greater (*p* always < 0.05) in suspensions from patients with RASopathies as compared to controls.

**Table 1:** EC_50_ for ADP, ristocetin, collagen, and AA for aggregation of PRP from controls and patients with RASopathies (mean ± SD of 2 different determinations)

Stimulus	PRP from controls	PRP from patients with RASopathies	PRP from patients with pathologic bleeding scores	PRP from patients with normal bleeding score
Adenosine diphosphate (ADP µ*M*)	1.84 ± 0.46	3.6 ± 1.2*	4.5 ± 1.1	3.23 ± 1.1
Ristocetin (mg/ml)	0.8 ± 0.3	1.1 ± 0.26	2 ± 0.3	1 ± 0.1
Collagen (µg/ml)	0.42 ± 0.13	0.95 ± 0.88*	2.5 ± 1	0.65 ± 0.3
Arachidonic acid (AA, m*M*)	0.30 ± 0.01	0.4 ± 0.19	1 ± 0.2	0.4 ± 0.1

*p < 0.05, significant difference between PRP of control individuals and that from patients with RASopathies

A 2-fold higher concentration of ADP was required to obtain aggregation of platelet from patients with RASopathies than the one needed  in controls. Collagen was also reduced in this patient setting. In addition, the maximal extent of aggregation in response to high concentrations of ADP (10 µM), collagen (10 µg/ml), and arachidonic acid (1 mM) was also reduced in patients with RASopathies when compared to controls (data not shown).

Table 2 shows clotting factors and aggregation data of patients with pathologic bleeding score.

**Table 2:** Patient’s bleeding score, clotting factor and aggregation data

Patient	Gene	Bleeding score*	Clotting factor (% of normal)				Platelet response to**			
			V	VII	X	XIII	ADP	Collagen	Ristocetin	AA
1	*SOS1*	7	67	48	64	47	2.8	0.8	1.1	0.4
2	*SOS1*	4	83.2	61	87	94.4	2.8	0.4	1.1	0.4
3	*SOS1*	3	55	81	76	73				
4	*PTPN11*	3	83	47.7	57	42	4	4	1.5	1
5	*PTPN11*	3	78.6	52	60	46	4	0.8	1.1	0.4
6	*PTPN11*	5	54	46	55		6	2	1.5	0.4
7	*PTPN11*	3	75	57	88	71	5	2	2	0.5
8	*BRAF*	3	85	58.5	74	29	1.2	0.4	1.1	0.4
9	*MAP2K1*	3	78	72			2	2	1.1	0.4
10	*PTPN11*	3	81	36	78	74	4	0.8	1.1	0.4
11	*PTPN11*	3	58	60.2	47.4		6	0.8	1.5	0.5
12	*RIT1*	3	50.2	46	50	50	5	0.8	1.5	
13	*RIT1*	3	55	44	50.5		5	1.2	1.2	0.5
14	*PTPN11*	3	117	86	62		4	0.4	1.1	0.4

*These data are expression of 3 evaluations per patient

**The response to platelet aggregation is the average of two repetitions

Patients with pathologic bleeding score showed higher prevalence of splenomegaly (*p* = 0.006).

Patients with pathologic BQ score showed lower levels of FV (74.9 ± 17 vs 93 ± 12, *p* = 0.001), FVII (57.8 ± 14 vs 73.7 ± 14, *p* = 0.003), FX (66.7 ± 13 vs 83.6 ± 12, *p* = 0.0008), FXIII (58.4 ± 21 vs 86.6 ± 20, *p* = 0.002), higher vWAg (85 ± 45 vs 60 ± 15, *p* = 0.04), and longer aPTT (38 ± 4.2 vs 33 ± 3.7, *p* = 0.04).

Platelet sensitivity to Ristocetin (4.5 ± 1.1 vs 3.23 ± 1.1; *p* = 0.01), and to collagen (2.5 ± 1.1 vs 0.65 ± 0.3; *p* = 0.03) was significantly lower in patients with pathologic PBQ score than patients with normal bleeding score (Table 1).

Correlation analysis showed that *PTPN11* patients with Noonan syndrome have higher prevalence of Factor XII deficiency (r = 0.56, *p* = 0.03). The presence of hematomas inversely correlated with factor V (r = − 0.5, *p* = 0.002), factor VII (r = − 0.4, *p* = 0.003), factor X (r = − 0.56, *p* = 0.002), and factor XIII (r = − 0.56, *p* = 0.004), and directly correlated with platelet response to collagen (r = 0.42, *p* = 0.02) and platelet response to AA (r = 0.44, *p* = 0.01). The presence of splenomegaly correlated with the presence of hematoma (r = 0.387, *p* = 0.006), platelet response to Ristocetin (r = 0.36, *p* = 0.04), and platelet response to AA (r = 0.35, *p* = 0.04) and inversely correlated with factor V levels (r = − 0.35, *p* = 0.03).

The two LS patients showed a slightly prolonged aPTT and normal platelet function.

## Discussion

RASopathies can be associated with an increased risk of bleeding and bruising, and a variety of bleeding abnormalities have been described in these disorders, but their causes still remain unclear.

The aim of the current study was to identify the frequency and type of bleeding disorders in patients with RASopathies, and to evaluate possible links with associated laboratory findings and genotype. Pathological bleeding score was recorded in almost half of the patients, regardless of the gene involved. No significant genotype–phenotype correlation was identified. The role of the small number of patients cannot be excluded. The mutational spectrum of patients with bleeding disorders was largely overlapping with that of the NS general population, including mutations in *PTPN11, SOS1, RIT1, BRAF*, and *MAP2K1* genes. Similar to unselected NS cohorts, *PTPN11* mutations were responsible for half of the cases. Another 30% of patients had mutations in *SOS1* and *RIT1*, whereas *BRAF* and *MAP2K1* mutations were identified in single families. None of the patients harboured mutations in *RAF1* and *LZTR1,* two genes frequently mutated in NS. Considering that bleeding disorders have been described in patients with mutations in both these genes [9, 27, 28], the absence of LZTR1 and RAF1 mutations in our patients is very likely related to the limited size of the study cohort rather than to specific genotype–phenotype correlations.

Possible genotype–phenotype correlations with coagulation defects in the context of RASopathies were initially hypothesized for *PTPN11* and *SOS1*. A study performed on 27 NS patients identified a *PTPN11* mutation in 21 and bleeding defects in 9, and therefore a correlation between *PTPN11* mutations and bleeding defects in the context of NS was hypothesized. To date, more than 20 genes responsible for RASopathies have been described and correlations with bleeding disorders cannot be excluded. However, despite the limited number of cases described in the studies reported so far, present results as well as those previously published seem to support the absence of significant correlations with bleeding defects in RASopathies.

Among the laboratory findings described in RASopathy patients with bleeding defects, factor XI deficiency and platelet abnormalities are most frequently described [10–20]. Patients with NS seem to have an imbalance in fibrinolytic components favouring fibrinolysis. This may be an important contributor to the bleeding [29]. Acquired cases of von Willebrand syndrome have been described and the destruction of the von Willebrand factor could explain bleeding in some NS patients with pulmonary valve stenosis [17]. A number of studies identified no correlation between coagulation study results and bleeding risk in NS [10–30]. In regard of laboratory findings, our RASopathy patients with bleeding disorders showed lower levels of FV, FX, FXIII coagulation factors, higher vWAg, longer aPTT, and lower platelet sensitivity to Ristocetin, AA and collagen. Moreover, they presented a higher prevalence of splenomegaly. Of note, the presence of hematomas was inversely correlated with factor V, factor VII, factor X and, factor XIII levels, whereas it was directly correlated with platelet response to collagen and platelet response to AA. Other significant correlations highlighted in this study was the observation that splenomegaly was directly correlated with the presence of hematomas, and to platelet response to Ristocetin and AA, but inversely correlated with factor V levels. It might be hypothesized that platelet dysfunction causes or is related to splenomegaly. On the basis of these data it might be suggested that the combination of platelet dysfunction and coagulation factor deficiency causes bleeding diathesis or, alternatively, that coagulation factor deficiency impair or worsen platelet function.

Although there is currently no consensus on the best strategy for bleeding disorder diagnosis in NS patients, it has been suggested that, at time of diagnosis of NS, a complete blood cell count (CBC) with differential and PT/aPTT should be performed. Then, in consultation with hematologist, FIX, FXI, and FXII levels, von Willebrand factor, and platelet aggregation should be investigated [31].

Recently, a significant reduction in platelet aggregation, induced by low concentrations of GPVI and CLEC-2 agonists, and a decrease in thrombus growth on a collagen surface under arterial shear stress were demonstrated in a NS mouse model carrying a gain-of-function mutation of SHP2. Conversely, LS-associated SHP2 loss-of-function results in increased platelet activation [25]. This study suggests the presence of thrombo-pathies linked to platelet signalling defects in NS.

Data obtained in the current study demonstrated that platelet aggregation in response to ADP and collagen stimulus was impaired in patients affected by RASopathy.

These results suggest that the hyperactivation of RAS-MAPK pathway interfere with different platelet-specific signalling pathway, thus confirming data obtained in mouse model.

In addition, the maximal extent of aggregation in response to high concentrations of ADP, collagen, and arachidonic acid was also reduced in patients with RASopathies as compared to controls. These data might suggest it is not only question of reduced sensitivity.

## Conclusions

Our findings underline that the combination of platelet dysfunction and coagulation factor deficiency is at the basis of haemostatic abnormalities in patients affected by RASopathies. Splenomegaly, frequently detected in these patients, might be a suggestive sign for platelet dysfunction.

Present and previous studies show that patients with RASopathies can experience multiple bleeding disorders, including platelet-related disorders. Therefore, comprehensive clinical evaluations should be carried out both at diagnosis and in follow-up, in particular if patients are symptomatic and before any surgical procedures. Since there is no current consensus on management of bleeding complications, it is important that physicians closely monitor these patients [9–11]. Further studies are needed to better evaluate these features and set up a specific follow-up.

## Data Availability

Data are available by request.

## Abbreviations

AA: Arachidonic acid
CFCS: Cardio-facio-cutaneous syndrome
LS: Leopard syndrome
MAPK: Mitogen activated protein kinase
NS: Noonan syndrome
NF1: Neurofibromatosis type 1
PBQ: Pediatric Bleeding Questionnaire
PT: Prothrombin Time
aPTT: Partial Thromboplastin Time
